# Intensive care social workers as simulated families for communication skills training

**DOI:** 10.5116/ijme.606a.f293

**Published:** 2021-04-27

**Authors:** Alex Yartsev

**Affiliations:** 1 Westmead Intensive Care Unit, Westmead Hospital, University of Sydney, Australia, Sydney, Australia

**Keywords:** End of Life Care, Communication, Simulation Training, Social Work, Critical Care

## To the Editor

Effective communication with grieving and distressed families in the Intensive Care Unit (ICU) is an essential part of a critical care practitioner's skill set. Simulation-based communication skills training for "difficult conversations" and "breaking bad news" is a well-established healthcare worker education strategy.^1^^,^^4^^,^^6^ However, such training strategies typically involve the use of trained actors or lay volunteers. Many studies of communication skills training^2^ explicitly state that their simulated patients were "highly trained, paid actors" as a limitation of their research, remarking that the prohibitive cost of using such simulated patients would place the program they designed well out of reach of resource-poor educators. Beyond the fiscal cost, maintaining a trained bank of lay actors is a non-trivial undertaking.^3^ Extensive briefing is required to familiarise lay actors with the context of end of life (EOL) discussions, matters relating to ICU care, brain death, organ donation, and the requirements of ICU medical trainees.

Fortunately, most large facilities already have an untapped resource of highly skilled communication specialists in the form of ICU-affiliated social workers. These allied health professionals are frequently exposed to every permutation of grief and coping behaviour. This experience grants them a unique perspective on the use of communication skills in these scenarios. Their involvement in communication training programs remains a promising underexplored territory. Published research on this topic is limited to a single study^5^ which reported positive responses to structured feedback from social workers in communication skills practice for Emergency Medicine trainees. Thus far, no investigator has explored the idea of leveraging the skills and experience of ICU social workers to simulate challenging communication scenarios.

This paper describes an educator's experience of involving experienced ICU social workers as the facilitators of a practical simulation-based communication training program for senior ICU medical trainees. This program was conducted in a large tertiary Intensive Care Unit with a group of senior ICU medical trainees, several dedicated social worker staff and a rotating roster of non-ICU social workers participating in clinical family conferences. The program consisted of quarterly simulation sessions in the form of family discussions, carried out with ICU social workers acting as simulated patient families. Typically, ten social worker staff and ten trainees participated. The sessions ran over 90 minutes (three 30-minute blocks), with three ICU trainees participating directly by taking part in the simulation. Each block consists of 5 minutes preparation time, ten minutes scenario run-time, and fifteen minutes of feedback. This program's unique element was that the scenarios were developed and scripted by the ICU social workers with some input from senior ICU medical staff. For each scenario, the ICU trainee is given a paragraph of clinical information to read during the preparation time. The trainee and social workers then engage in a simulated family discussion observed by the rest of the participants and faculty. At the end of the scenario, all parties are invited to participate in the debrief discussion; emphasis was placed on the feedback from the social workers participating as simulated family members, some of whom choose to give feedback in-character.

The program coordinator collected feedback regarding this training program from the participants through a semi-structured interview process. The responses were remarkably positive, particularly around the themes of authority and participant validation. Authority, or the possession of fundamental expertise and resources to evaluate communication performance, was identified as a recurring theme through all of the participant interviews. The ICU social workers felt very comfortable in their roles as simulated families. They were enthusiastic about giving feedback to the medical staff, mainly because they felt that their skill set gave them the authority to do so. Among the senior ICU trainees, there was a clear undercurrent of profound respect for the skill set of the ICU social workers. This manifested in many ways, but the most illustrative example is the fact that several of them had previously sought out social workers independently to help them practice communication skills or asked for direct feedback regarding their performance following difficult interactions with patients or families. This is similar to the findings of the previous authors,^5^ who explored the influence of social worker feedback on observed family interactions with Emergency Department medical staff. In that study, social workers' formative feedback and advice were viewed as highly influential by most of the residents, at or above the level of significance attributed to the advice and feedback from a senior clinician.

Participant validation, or the mutual appreciation of value, was a dominant theme in the feedback. The two groups of staff expressed how much they valued teaching others' participation in the project. Social workers derived a sense of satisfaction from being able to contribute to the education of medical staff, which made the program more sustainable and improved the recruitment of key resource staff. Participation in this program was sufficiently rewarding for the social workers that the coordinators were inundated with willing faculty.

In conclusion, the use of intensive care social workers promises to be an effective and sustainable method of conducting a practical communication skills training program.  Participant feedback suggests that ICU social workers are acknowledged as having authority over communication skills teaching, and their feedback is highly respected by senior ICU trainees. The sustainability of the program was supported by a high level of interdisciplinary respect between ICU social workers and medical staff. This experience has implications for the design of communication skills education programs, as it suggests that ICU social workers should have increased involvement in communication skills training. Further studies are warranted to determine the influence of this program design on trainee participation, trainee skill acquisition and communication performance.

### Conflict of Interest

The author declares that he has no conflict of interest.
